# Unveiling Insights: A Comprehensive Review of the Role of Medical Thoracoscopy in Pleural Effusion Assessment

**DOI:** 10.7759/cureus.53516

**Published:** 2024-02-03

**Authors:** Anjana Ledwani, Babaji Ghewade, Ulhas Jadhav, Sameer Adwani, Pankaj Wagh, Ashwin Karnan

**Affiliations:** 1 Respiratory Medicine, Jawaharlal Nehru Medical College, Wardha, IND

**Keywords:** comparative analysis, therapeutic interventions, clinical applications, diagnostic modalities, pleural effusion assessment, medical thoracoscopy

## Abstract

Pleural effusion, characterized by abnormal fluid accumulation in the pleural cavity, poses diagnostic and therapeutic challenges across various medical conditions. This comprehensive review explores the role of medical thoracoscopy in assessing pleural effusions, providing insights into its historical context, procedural intricacies, diagnostic performance, safety considerations, and clinical applications. Medical thoracoscopy, a minimally invasive endoscopic procedure, offers advantages such as high diagnostic yield, therapeutic interventions, real-time assessment, and a minimally invasive nature. The review critically analyzes the procedure's advantages and disadvantages, including technical expertise, risk of complications, resource intensity, and patient selection criteria. Comparative analyses with alternative diagnostic modalities highlight the unique benefits of medical thoracoscopy in specific clinical scenarios. The diagnostic yield of medical thoracoscopy is examined, considering sensitivity and specificity in various contexts. Patient selection criteria, complications, and safety measures are discussed, emphasizing the importance of careful consideration in integrating thoracoscopy into clinical practice. The review further explores its clinical applications, including differentiating exudative and transudative effusions, identifying specific etiologies, and its role in treatment planning. In conclusion, medical thoracoscopy emerges as a valuable tool in the comprehensive management of pleural effusions, offering a nuanced approach to diagnosis and treatment. The evolving landscape of diagnostic modalities underscores the continued significance of medical thoracoscopy and potential advancements in the field.

## Introduction and background

Pleural effusion refers to the abnormal accumulation of fluid in the pleural cavity, the space between the layers of the pleura surrounding the lungs. This condition can arise from various underlying causes, such as infections, malignancies, heart failure, or inflammatory disorders. Understanding the nature and composition of pleural effusions is crucial for accurate diagnosis and effective management [1]. The assessment of pleural effusion holds paramount clinical importance due to its association with a broad spectrum of medical conditions. Timely and accurate diagnosis of the etiology of pleural effusion is critical for appropriate treatment planning and patient care. Neglecting the evaluation of pleural effusions may result in delayed or inadequate management, leading to increased morbidity and mortality [2].

This review aims to critically examine the role of medical thoracoscopy in assessing pleural effusion. By exploring the historical context, procedural intricacies, diagnostic performance, safety considerations, clinical applications, and future directions for medical thoracoscopy, this review aims to provide a thorough understanding of its place in the diagnostic armamentarium. Through a structured analysis, we aim to shed light on how medical thoracoscopy complements and, in specific scenarios, surpasses other diagnostic modalities in the evaluation of pleural effusions.

## Review

Medical thoracoscopy: an overview

Definition and Explanation

Medical thoracoscopy, also known as pleuroscopy, is a minimally invasive endoscopic procedure utilized by pulmonologists to evaluate, diagnose, and treat pleural pathologies, mainly pleural effusions [3-4]. The procedure can be diagnostic or therapeutic and is commonly performed under local anesthesia or using non-disposable rigid or semi-flexible (semi-rigid) instruments [5]. Medical thoracoscopy is a safe and effective modality for diagnosing and treating several pleuro-pulmonary diseases [5]. It is less invasive and less expensive compared to surgical thoracoscopy, which is better termed "video-assisted thoracic surgery" (VATS) and is performed in an operating room [5]. The procedure can be performed in an endoscopy suite and is easier to learn than flexible bronchoscopy. In many European countries, medical thoracoscopy is already a part of the respiratory training program [5].

Indications for Medical Thoracoscopy

Undiagnosed pleural effusion: Medical thoracoscopy emerges as a crucial diagnostic tool when faced with a pleural effusion of unknown origin. In instances where initial investigations fail to pinpoint the underlying cause, this procedure offers a distinctive advantage. By providing direct visualization and the potential for biopsy, medical thoracoscopy enables clinicians to delve into the pleural space, aiding in identifying elusive etiologies. This targeted approach enhances diagnostic accuracy and facilitates timely intervention and appropriate patient management [6].

Malignant pleural diseases: The role of medical thoracoscopy extends significantly in malignant pleural diseases. Beyond its diagnostic utility, this procedure is a cornerstone in the staging and assessment of pleural malignancies. By allowing direct visualization, medical thoracoscopy aids in evaluating the extent of tumor involvement. Furthermore, acquiring tissue samples through this technique is instrumental for histopathological examination, essential in confirming the presence of malignancy and guiding subsequent therapeutic decisions. Medical thoracoscopy in the oncological context underscores its pivotal role in advancing diagnostic precision and tailored treatment strategies [6].

Suspected infections: Medical thoracoscopy is a valuable resource in cases where pleural infections are suspected. This procedure facilitates the identification of infectious agents by providing a direct and visual inspection of the pleural space. This direct visualization is particularly advantageous in selecting appropriate antimicrobial treatment strategies. The insights gained from medical thoracoscopy contribute to accurate diagnosis and targeted therapeutic interventions, ultimately improving patient outcomes in cases of pleural infections [6].

Pleurodesis: Beyond its diagnostic capabilities, medical thoracoscopy finds application in therapeutic interventions, notably in pleurodesis. This procedure involves the introduction of substances into the pleural space to induce adhesion between the pleural layers. This adhesive effect serves as a preventive measure against recurrent effusions. With its real-time visualization and precise delivery capabilities, medical thoracoscopy is preferred for pleurodesis. This dual diagnostic and therapeutic role positions thoracoscopy as a versatile procedure in managing pleural effusions, offering a comprehensive solution to address the underlying cause and its symptomatic manifestations [6].

Evaluation of pleural thickening: Thoracoscopy is a valuable tool in assessing pleural thickening, crucial in distinguishing between benign and malignant causes. Through direct visualization, this procedure allows clinicians to examine the characteristics and nature of pleural thickening, providing essential information for accurate diagnosis and treatment planning. Differentiating between benign and malignant thickening is vital for prognostication and guiding appropriate therapeutic strategies. Medical thoracoscopy, with its capacity for real-time inspection, contributes significantly to the nuanced evaluation of pleural thickening, enhancing the precision of diagnostic outcomes [6].

Advantages and disadvantages

Advantages

High diagnostic yield: Medical thoracoscopy is a powerful diagnostic tool, distinguished by its ability to visualize the pleura directly. This attribute translates into a high diagnostic yield, particularly in scenarios where traditional diagnostic methods may encounter limitations. The direct access to the pleural space afforded by thoracoscopy facilitates a detailed examination, enabling healthcare professionals to uncover elusive etiologies and contributing significantly to the precision of diagnostic outcomes. The enhanced diagnostic yield positions medical thoracoscopy as a valuable and often indispensable component of the diagnostic armamentarium, especially in cases where conventional approaches fall short [7].

Therapeutic interventions: Beyond its diagnostic prowess, medical thoracoscopy stands out for its versatility in facilitating therapeutic interventions. The procedure offers a dynamic platform for interventions such as pleurodesis or targeted biopsies, transforming it into a dual-purpose tool for managing pleural diseases. This therapeutic dimension is particularly advantageous, allowing clinicians to identify the underlying cause and directly address and mitigate the consequences of pleural pathologies. The integration of diagnostic and therapeutic capabilities underscores the multifaceted utility of medical thoracoscopy in optimizing patient care and outcomes [7].

Real-time assessment: A distinctive feature of medical thoracoscopy is its capacity for real-time assessment of the pleural space. This real-time capability empowers healthcare professionals to make immediate decisions based on visual findings during the procedure. The ability to dynamically evaluate the pleura enhances diagnostic precision and allows for prompt adjustments to the course of action, ensuring a more agile and responsive approach to patient care. Real-time assessment makes medical thoracoscopy an invaluable tool for clinical decision-making, particularly when timely interventions can significantly impact patient outcomes [7].

Minimally invasive: Medical thoracoscopy is minimally invasive, presenting a notable departure from traditional open procedures. This characteristic translates into tangible patient benefits, including reduced postoperative pain, shorter hospital stays, and quicker recovery. The minimally invasive nature of thoracoscopy aligns with contemporary trends in patient-centered care, emphasizing improved patient comfort and a faster return to normal activities. As a result, medical thoracoscopy enhances diagnostic and therapeutic capabilities and contributes to an overall positive patient experience [7].

Disadvantages

Technical expertise: The performance of thoracoscopy necessitates a high level of technical expertise and specialized training, contributing to a potential limitation in its widespread adoption across various healthcare settings. The intricate nature of the procedure, involving the insertion of a thoracoscope into the pleural space, demands a skill set that may only be readily available in some medical environments. This requirement for specialized expertise underscores the importance of targeted training programs to ensure that healthcare professionals possess the proficiency needed to execute medical thoracoscopy effectively. The level of technical expertise required becomes a critical factor influencing the accessibility of this procedure within the broader healthcare landscape [7].

Risk of complications: While medical thoracoscopy is generally considered a safe procedure, it is not exempt from risks, and clinicians must be cognizant of potential complications. These complications may include pneumothorax, bleeding, infection and pain. Though these adverse events are infrequent, their occurrence underscores the need for careful patient selection and the importance of skilled practitioners. Vigilance in managing potential complications is crucial for ensuring the safety of patients undergoing thoracoscopy. Balancing the procedure's potential benefits with the associated risks becomes imperative in decision-making, emphasizing the need for a comprehensive risk-benefit assessment by healthcare providers [7].

Resource intensive: The resource-intensive nature of medical thoracoscopy adds another layer of consideration. The procedure requires specific equipment and facilities, contributing to its potential limitation in accessibility. The need for specialized instruments, including the thoracoscope and associated tools, may pose challenges in healthcare settings with limited resources or infrastructure. This resource demand necessitates a strategic approach to integrating thoracoscopy into clinical practice, ensuring that the requisite resources are available to support the safe and effective implementation of the procedure [7].

Contraindications: Certain patient conditions, such as severe cardiopulmonary compromise, may be contraindications to thoracoscopy. This highlights the importance of careful patient selection based on individual health profiles. Patients with pre-existing conditions that pose a heightened risk during the procedure may need alternative diagnostic or therapeutic approaches. Recognizing contraindications and assessing patient suitability become critical aspects of the decision-making process, influencing the appropriateness of medical thoracoscopy in specific clinical scenarios [7].

Diagnostic yield of medical thoracoscopy

Sensitivity and Specificity

The sensitivity and specificity of medical thoracoscopy in diagnosing pleural effusions have been reported in several studies. The diagnostic yield of medical thoracoscopy varies across different investigations, with sensitivity and specificity rates being influenced by the underlying causes of the effusion. In studies conducted in India, the diagnostic yield of pleuroscopy ranged from 67% to 97% in cases of undiagnosed pleural effusions [8]. A one-year prospective study investigating the diagnostic yield of medical thoracoscopy for undiagnosed exudative pleural effusions reported varying sensitivity and specificity rates, highlighting the influence of different factors on the procedure's effectiveness [9]. Medical thoracoscopy has been recognized as a valuable diagnostic tool, particularly in cases of malignant pleural disease, with reported diagnostic yields of up to 95% [10]. The procedure is considered a gold standard for diagnosing and treating pleural diseases, offering high diagnostic yield in various contexts, including cases of undiagnosed exudative pleural effusions [10]. The sensitivity and specificity of medical thoracoscopy in diagnosing pleural effusions vary across studies and are influenced by factors such as the underlying cause of the effusion. While some studies report high diagnostic yields, it is essential to consider the specific clinical context when evaluating the effectiveness of medical thoracoscopy [8-10].

Comparative Analysis With Other Diagnostic Tools

Clinical history and physical examination: The initial step in diagnosing pleural effusions often involves a thorough clinical history and physical examination. While these methods serve as the frontline diagnostic approach, their accuracy may diminish in cases of complex or atypical presentations. Clinical history and physical examination provide essential insights into a patient's health. Still, their limitations underscore the need for supplementary diagnostic tools, particularly in scenarios where a definitive diagnosis remains elusive [11].

Imaging techniques: Various imaging modalities, including ultrasound, computed tomography (CT), and magnetic resonance imaging (MRI), play a crucial role in assessing pleural effusions. These techniques offer valuable information regarding the presence and potential causes of effusions. However, they may only sometimes suffice for a definitive diagnosis, emphasizing the importance of combining imaging findings with other diagnostic methods for a more comprehensive understanding of the underlying pathology [11].

Thoracentesis: Thoracentesis, involving fluid drainage from the pleural cavity, is a diagnostic procedure that aids in confirming the presence of pleural effusion. While it provides confirmation, it may need to improve when it comes to identifying the precise cause of the effusion. The limitations of thoracentesis underscore the necessity for complementary diagnostic approaches to uncover the underlying etiology [11].

Percutaneous pleural biopsy: Percutaneous pleural biopsy, utilizing a needle to obtain a sample of the pleural tissue, offers a pathway to a definitive diagnosis. However, its accuracy may be compromised, especially in cases of malignant pleural disease. When sensitivity and specificity become crucial, medical thoracoscopy emerges as a more accurate alternative, with reported values reaching up to 95% for malignant pleural disease [10].

Combined approach with pleural fluid analysis: A combined approach that integrates pleural fluid analysis, such as adenosine deaminase (ADA) and protein levels, with additional tests like closed needle biopsy, histology, and culture presents a comprehensive diagnostic strategy. This approach demonstrates high sensitivity (around 93%) and specificity (100%), particularly in regions with a high incidence of tuberculosis (TB). It is a cost-effective alternative to medical thoracoscopy, providing accurate and efficient diagnostic outcomes [12].

Patient Selection Criteria

Clinical history: A detailed exploration of the patient's clinical history is a foundational element in the decision-making process for medical thoracoscopy. The evolution of the disease, whether acute or chronic, can offer critical insights into its nature and progression. This information becomes pivotal in determining the appropriateness of medical thoracoscopy as a diagnostic or therapeutic intervention. For instance, chronic conditions may necessitate a different approach than acute presentations, and a comprehensive clinical history aids in tailoring the procedure to the specific characteristics of the pleural effusion [5].

Physical examination findings: The findings from a thorough physical examination provide valuable data for assessing pleural effusions. Clinical signs such as increased vocal fremitus, decreased vocal resonance, and diaphoresis can indicate pleural pathology; these physical markers aid in identifying and characterizing pleural effusions, providing crucial information for decision-making. Incorporating physical examination findings into the patient evaluation procedure ensures a multi-faceted approach, allowing for a more nuanced understanding of the clinical presentation and guiding subsequent diagnostic or therapeutic interventions [5].

Contraindications: A clear identification of contraindications is imperative to ensure patient safety during medical thoracoscopy. Patients with severe chronic obstructive pulmonary disease (COPD), respiratory insufficiency, hypoxemia, and hypercapnia may be at increased risk. They may not tolerate the induction of a pneumothorax without compromising gas exchange. Additionally, those with fever, unstable cardiovascular status, or recent myocardial infarction are considered contraindicated for the procedure. Recognition of these contraindications is vital in preventing potential complications and adverse outcomes, guiding clinicians in appropriately selecting patients for medical thoracoscopy [5,13].

Adequate training of thoracoscopists: Thoracoscopist proficiency plays a pivotal role in the success and safety of medical thoracoscopy. Adequate training in both cognitive understanding and technical skills is essential. This training ensures that the thoracoscopist can precisely navigate the procedure, minimizing the risk of complications. A well-trained thoracoscopist contributes to the overall efficacy of the procedure and patient outcomes, emphasizing the importance of ongoing education and training in this specialized field [5].

Minimum number of procedures: To enhance procedural expertise and familiarity with instrumentation, a minimum of 20 procedures is recommended. This threshold reflects the importance of hands-on experience in optimizing the thoracoscopist's skills. Achieving this level of proficiency ensures competence that contributes to the safety and effectiveness of medical thoracoscopy. The emphasis on a minimum number of procedures underscores the significance of practical experience in mastering the intricacies of this diagnostic and therapeutic technique [5].

Complications and safety measures

Common Complications

Pain: Post-thoracoscopic pain emerges as a common complication, affecting approximately 32.74% of patients undergoing medical thoracoscopy. This pain is a natural consequence of the procedure and can challenge patient comfort and recovery. Management strategies for post-thoracoscopic pain may include analgesics and other pain control measures. The prevalence of pain underscores the importance of proactive pain management protocols to enhance patient satisfaction and overall procedural experience [14].

Transient air leak: In about 11.61% of cases, transient air leaks represent a manageable complication associated with medical thoracoscopy. In most instances, conservative measures are effective in addressing this issue. The transient nature of the air leak suggests that, while it may contribute to postoperative discomfort, it typically does not pose a significant threat to patient well-being. Adequate patient education and monitoring contribute to successfully managing transient air leaks following thoracoscopy [14].

Fever: Fever, identified as a complication in 3.9% of cases, highlights the inflammatory response associated with medical thoracoscopy. While fever may be common, close monitoring is essential to differentiate between expected postoperative fever and potentially concerning systemic infections. Timely identification and appropriate management contribute to minimizing the impact of fever on patient recovery [14].

Wound infection: Although rare, wound infections can occur as complications of medical thoracoscopy. Attention to aseptic techniques during the procedure and meticulous postoperative care are essential preventive measures. Prompt recognition and intervention in cases of wound infection are crucial to minimize the risk of further complications and ensure optimal patient outcomes [5].

Empyema: Empyema, though a rare complication, underscores the importance of vigilance in postoperative monitoring. This serious condition, characterized by the collection of pus in the pleural space, necessitates prompt diagnosis and intervention. Strategies to prevent empyema include meticulous infection control practices and close observation for any signs of postoperative infection [5].

Subcutaneous emphysema: Subcutaneous emphysema, occurring in 0.8% of cases, signifies the presence of air in the subcutaneous tissues. While typically a self-limiting condition, it highlights the importance of monitoring for any signs of respiratory distress and implementing appropriate measures if necessary. Patient education on expected postoperative changes can contribute to their understanding and reassurance regarding this transient complication [15].

Re-expansion pulmonary edema: Re-expansion pulmonary edema, although infrequent, is a severe complication that can occur, particularly in patients with pre-existing respiratory conditions. This underscores the need for careful patient selection, considering individual risk factors. Preoperative assessment and optimization of respiratory status contribute to minimizing the likelihood of this complication [5].

A comprehensive approach to mitigating complications involves careful patient selection, ensuring thoracoscopists are adequately trained, and adhering to established surgical techniques and protocols. Early identification and appropriate management of complications contribute to medical thoracoscopy's overall safety and effectiveness, ensuring favorable outcomes for most patients [16].

Strategies for Complication Prevention

Complications during thoracoscopy can be minimized by following strict standards of operation [6]. Choosing the position carefully at the outset with available radiographs, computed tomography, and ultrasound can help prevent complications [17]. Lung laceration is the most serious complication of medical thoracoscopy, but it can be managed well [15,16]. Hospital-acquired infections (HAIs) are low-risk and can be controlled by medical treatment [16]. Using a one-off dose of intravenous broad-spectrum antibiotics at the time of the procedure may also help prevent infections [16]. Medical thoracoscopy is generally considered a safe procedure, and the rates of complications such as empyema, pneumonia, and one-month mortality compare favorably with other large thoracoscopy series [16].

Management of Complications

Lung lacerations: During a lung laceration during thoracoscopy, prompt and targeted intervention is crucial. The initial step involves compressing the bleeding point utilizing the nearby lung tissue. This compression technique aims to control and stop the bleeding effectively. Additionally, a hemostatic agent, such as Tachosil®, can be employed to aid in the further cessation of bleeding. Managing lung lacerations requires a coordinated and rapid response to minimize the potential consequences and ensure patient safety [18].

Pulmonary complications: Pulmonary complications, including hypoxemia, hypercapnia, re-expansion pulmonary edema, and atelectasis, necessitate a comprehensive approach aligned with established medical protocols and guidelines. Management strategies may involve interventions such as supplemental oxygen therapy, mechanical ventilation if required, and vigilant monitoring of respiratory parameters. Tailoring the approach to the specific nature of the pulmonary complication is essential for optimizing patient outcomes and preventing further respiratory compromise [19].

Hospital-acquired infections: In cases where HAIs pose a risk, immediate medical treatment is employed for control. Prophylactic measures can include the administration of a one-off dose of intravenous broad-spectrum antibiotics at the time of the procedure. This preemptive approach minimizes the risk of infections associated with the thoracoscopic intervention. The judicious use of antibiotics aligns with infection control principles, preventing HAIs and promoting patient safety [16].

Prevention of complications: The prevention of complications in thoracoscopy is paramount and begins with strict adherence to established standards of operation. This includes meticulous attention to patient positioning, ensuring optimal access and visibility for the procedure. Available imaging techniques further enhance precision and minimize the risk of adverse events. By prioritizing preventive measures and following standardized protocols, healthcare professionals can significantly reduce the likelihood of complications during thoracoscopy. This proactive approach aligns with the principles of patient safety and procedural excellence [6].

Clinical applications

Differentiating Exudative and Transudative Effusions

Medical thoracoscopy is a valuable and minimally invasive procedure with dual applications in diagnostic and therapeutic realms for various pleural diseases [20]. Its pivotal role is particularly pronounced in the management of pleural effusions, which can be broadly classified into two categories: exudative and transudative effusions. This distinction is crucial as it informs treatment decisions and therapeutic strategies.

Exudative effusions: Exudative pleural effusions, often attributed to inflammation or infection, can be effectively diagnosed and characterized through medical thoracoscopy [15]. The procedure facilitates direct visualization of the pleura, allowing for site-directed biopsy and examination of the visceral pleura. This targeted approach enhances diagnostic precision, enabling healthcare professionals to identify the underlying cause of the exudative effusion. The insights gained from thoracoscopy contribute significantly to formulating appropriate treatment plans, emphasizing the procedure's role in both diagnosis and therapeutic decision-making [11].

Transudative effusions: Transudative pleural effusions, arising from hydrostatic or colloid osmotic pressure disruptions, result in fluid accumulation within the pleural space [13]. Medical thoracoscopy proves instrumental in differentiating between transudative and exudative effusions, which exhibit distinct clinical manifestations and necessitate varied treatment approaches. The ability of thoracoscopy to discern between these categories is essential for tailoring interventions to specific etiology, thereby optimizing patient care [13].

Diagnostic value: The diagnostic value of medical thoracoscopy surpasses conventional methods such as fluid cytology and 'blind' closed needle pleural biopsy [15]. The procedure's superiority lies in its capacity to provide direct visualization of the pleura, enabling precise diagnosis and differentiation between various pleural effusions. This diagnostic superiority is instrumental in determining the cause of the effusion and guiding clinicians toward appropriate and targeted treatment strategies. Medical thoracoscopy, therefore, stands out as a highly effective diagnostic tool, offering valuable insights for informed clinical decision-making [15].

Safety: Medical thoracoscopy is generally considered a safe procedure with rare complications [15]. Maintaining safety standards is paramount, and healthcare professionals can minimize the risk of adverse events by adhering to established protocols and taking necessary precautions during the procedure [17]. The rarity of complications underscores the procedure's safety profile, further reinforcing its viability as a valuable tool in managing pleural effusions.

Medical thoracoscopy emerges as a significant player in the differentiation between exudative and transudative pleural effusions, playing a crucial role in guiding treatment decisions and providing essential diagnostic information. Its diagnostic superiority, safety, and therapeutic applications position medical thoracoscopy as a valuable and versatile tool in the comprehensive management of pleural effusions [13,15,20].

Identification of Specific Etiologies

Medical thoracoscopy, also known as pleuroscopy, is crucial in identifying specific etiologies of pleural effusions. Pleural effusions have a wide range of causes, with congestive heart failure, pneumonia, malignancy, and pulmonary embolism being the most common [2,21]. When the etiology of a pleural effusion remains unclear, thoracoscopy can be employed to obtain pleural fluid for chemical, microbiological, and cytological analysis, providing valuable information about the underlying disease process [22]. Additionally, medical thoracoscopy with pleural biopsy has been shown to establish a diagnosis in more than 90% of cases, particularly in idiopathic and malignant pleural effusions [17]. This procedure allows for direct visualization of the pleura and targeted biopsy, identifying specific etiologies such as malignancy or inflammatory conditions [17]. Therefore, medical thoracoscopy is a valuable tool in the comprehensive management of pleural effusions, especially when the etiology is uncertain.

Role in Treatment Planning

Medical thoracoscopy plays a vital role in treatment planning for pleural effusions, particularly in cases where the etiology is unclear or complicated. Thoracoscopy can be used for diagnostic and therapeutic purposes, allowing for direct visualization of the pleura and targeted biopsy to aid in identifying specific etiologies such as malignancy or inflammatory conditions [13]. In complicated parapneumonic effusions, rigid thoracoscopy can help break up locations under direct vision, facilitating complete clearance [23]. Additionally, thoracoscopy can be used for therapeutic purposes, such as talc pleurodesis, a safe and efficient procedure for managing patients with malignant pleural effusions [13]. Medical thoracoscopy is a valuable tool in the comprehensive management of pleural effusions, identifying specific etiologies and guiding appropriate treatment planning [23].

Comparison with alternative diagnostic modalities

Thoracentesis

Thoracentesis is typically the initial invasive procedure to diagnose pleural effusions, as many laboratory tests can be run on the pleural fluid. It can establish the etiology of pleural effusion in approximately 75% of cases, with a diagnostic yield for malignant pleural effusion of 60% with the first pleural tap, increasing by up to 27% with the second tap. However, if no discrete areas of abnormalities are seen, the preferred next step is pleuroscopy or medical thoracoscopy, which has a diagnostic yield of over 90% [24]. Medical thoracoscopy is gaining popularity for diagnosing and treating exudative pleural effusions that remain undiagnosed after thoracentesis. It has a diagnostic yield of over 90% and is particularly useful in cases where thoracentesis has been non-diagnostic [13,24]. Additionally, thoracoscopy can be used for the therapeutic management of malignant pleural effusion, such as pleurodesis, and is generally well tolerated with a low mortality rate and few contraindications [11,13,25]. Alternative diagnostic modalities to thoracentesis include image-guided closed biopsies, such as ultrasound-guided pleural biopsy, which are minimally invasive alternatives with high diagnostic yields [26]. However, when thoracentesis is non-diagnostic, pleuroscopy or medical thoracoscopy is often the preferred next step due to its high diagnostic yield and therapeutic applications [24].

Closed Pleural Biopsy

Yield and diagnostic value: Closed pleural biopsy has historically been a diagnostic tool for pleural TB, offering a reasonable diagnostic yield. However, the advent of medical thoracoscopy has revealed a notable improvement in diagnostic efficacy, coupled with a lower complication rate compared to closed pleural biopsy [27]. Medical thoracoscopy's superior diagnostic yield is particularly advantageous in cases where a more accurate and comprehensive diagnosis is essential, contributing to its increasing preference in clinical practice.

Limitations and considerations: Closed pleural biopsy has limitations and associated considerations. One notable limitation is its inability to target specific areas of abnormal pleural thickening or nodularity, potentially leading to sampling errors and diagnostic challenges. Additionally, the procedure carries a risk of complications, such as pneumothorax, which can further impact patient outcomes [28]. Recognizing these limitations, closed pleural biopsy is now more selectively applied, especially in cases where alternative methods, such as medical thoracoscopy or image-guided biopsies, are deemed more suitable and are available.

Relevance in current practice: While closed pleural biopsy has not been entirely replaced, its role has evolved in the current medical practice. It is now considered when more advanced methods, such as image-guided pleural biopsy or thoracoscopy, are either unfeasible or unavailable [29]. When medical thoracoscopy is not viable, closed pleural biopsy may still be performed to obtain pleural tissue for diagnostic purposes. Despite the advent of alternative diagnostic modalities, closed pleural biopsy maintains its relevance in certain circumstances, particularly in resource-poor settings where access to more sophisticated techniques may be limited [27,30]. The procedure's continued relevance underscores its value in providing diagnostic insights when alternative approaches are constrained.

Imaging Techniques (CT Scan, Ultrasound)

CT scan: A CT scan combines a series of X-ray images taken from various angles with computer processing technology to create detailed images of internal organs, bones, soft tissue, and blood vessels [31-33]. CT scans help determine tumors' exact size and location and guide interventions such as biopsies. They are also used to diagnose acute injuries and conditions or chronic vascular conditions [31].

Ultrasound: Ultrasound imaging uses high-frequency sound waves to create a live video feed image of the inside of the body [31]. It is beneficial in evaluating the structures of joints within the body and can show the movement of the body's internal organs and blood flowing through blood vessels [31]. Ultrasound is also used to diagnose gallbladder disease, breast lumps, genital/prostate issues, joint inflammation, and blood flow problems and to monitor pregnancy [34].

Relevance in pleural effusion assessment: CT scans help identify the presence of pleural effusions and determine their size and location [33]. They can also help identify the underlying cause of the effusion, such as malignancy or infection [33]. Ultrasound is often used to guide thoracentesis, a procedure used to obtain pleural fluid for diagnostic purposes [31]. It can also be used to evaluate the pleural space and identify the presence of pleural thickening or nodularity. CT scans and ultrasound are helpful imaging modalities in evaluating pleural effusions [33]. CT scans are particularly useful in identifying the underlying cause of the effusion, while ultrasound is often used to guide diagnostic procedures such as thoracentesis [31,33].

Future directions and research opportunities

Emerging Technologies in Pleural Effusion Diagnosis

Imaging techniques: Advances in imaging techniques, particularly magnetic resonance imaging and computed tomography, represent significant strides in enhancing diagnostic capabilities when it comes to pleural effusions [35,36]. The improved image quality afforded by these technologies enables healthcare professionals to visualize pleural abnormalities with greater clarity and precision. These imaging modalities are pivotal in diagnosing and characterizing pleural effusions, contributing to more accurate and targeted treatment strategies. Integrating advanced imaging techniques has become integral to the contemporary diagnostic approach, allowing for a comprehensive evaluation of pleural diseases.

Computer-aided diagnostic analysis: Computer-aided diagnostic analysis has revolutionized the field of pleural diseases, specifically identifying cancer cells in pleural fluid [35]. Deep learning algorithms, trained with CT image data, demonstrate promising adequacies in discerning characteristics and diagnostic criteria for pleural effusion diagnosis. This technological innovation holds the potential to augment the accuracy and efficiency of diagnostic processes, providing valuable insights that can guide clinical decision-making. Integrating computer-aided diagnostic tools represents a cutting-edge approach for more precise and reliable diagnostic outcomes in pleural diseases.

Handheld ultrasound: Handheld ultrasound has emerged as a rapid and accurate alternative for diagnosing pleural effusions, offering advantages such as portability and real-time imaging without the need for ionizing radiation, as required in chest X-rays [37]. This technology has demonstrated reliability in measuring and quantifying pleural effusions, providing clinicians with a valuable point-of-care tool for efficient and non-invasive assessment. The adoption of handheld ultrasound contributes to diversifying diagnostic options, particularly in settings where immediate bedside evaluations are essential for timely decision-making and patient care.

Future research: Future research directions in diagnosing and treating pleural diseases may explore a range of emerging technologies. This includes risk stratification for patients with pleural diseases, non-surgical treatment options, and the utilization of advanced technologies such as 3D high-definition video technology and robotic-assisted thoracic surgery [11,36]. Investigating the potential of these technologies holds promise for optimizing diagnostic accuracy, refining treatment approaches, and minimizing invasiveness in some instances. The incorporation of cutting-edge technologies underscores the dynamic nature of advancements in the field, providing avenues for continuous improvement in patient outcomes and the overall management of pleural diseases.

Potential Improvements in Medical Thoracoscopy

Technological advancements: Technological advancements have significantly enriched thoracoscopy capabilities, introducing tools such as autofluorescence, narrow-band imaging, and infrared light. These technologies enhance the diagnostic precision of thoracoscopic procedures [38]. Autofluorescence, for example, allows for improved visualization of abnormal tissues by exploiting the differences in fluorescence between normal and abnormal cells. Narrow-band imaging and infrared light enhance contrast and detailed imaging of the pleural surface. Integrating these technological features represents a progressive step in refining the diagnostic potential of thoracoscopy, aiding clinicians in more accurately identifying and characterizing pleural pathologies.

Semi-rigid thoracoscopy: Adopting semi-rigid thoracoscopy is a noteworthy advancement, offering enhanced maneuverability within the pleural space. This approach is characterized by a smaller incision site and potentially lower anesthetic requirements, contributing to its appeal as a less invasive alternative [39]. The promising diagnostic yield and low rate of significant complications associated with semi-rigid thoracoscopy position it as a valuable option in the diagnostic armamentarium. The maneuverability advantage and comparable diagnostic outcomes underscore the potential for semi-rigid thoracoscopy to become a preferred choice in specific clinical scenarios.

Comparative studies: Comparative studies between semi-rigid and rigid thoracoscopy have yielded intriguing findings. Despite the generally larger biopsy specimens obtained through rigid thoracoscopy, small-scale trials have suggested that both approaches exhibit a comparable diagnostic yield [39]. This finding holds particular significance in the contemporary landscape, where the adequacy of tissue samples is paramount for molecular testing and participation in clinical trials. The potential equivalency in diagnostic outcomes contributes to the ongoing discourse regarding selecting the most appropriate thoracoscopic approach based on patient characteristics and procedural considerations.

Cost-effectiveness: A notable aspect of thoracoscopy, mainly when employed as an intermediate drainage procedure between tube thoracostomy and VATS, is its significant cost-effectiveness [40]. This becomes particularly relevant in healthcare settings where optimizing resource utilization is crucial. Medical thoracoscopy can provide a cost-efficient alternative by avoiding the need for surgical thoracoscopy under general anesthesia. Continued research into the cost-effectiveness of different thoracoscopy approaches provides valuable insights for healthcare decision-makers and practitioners, influencing the broader integration of these techniques into routine clinical practice.

Areas for Further Investigation

Optimal timing of medical thoracoscopy: Future studies are essential to evaluate the optimal timing of medical thoracoscopy in managing pleural infections and its potential role in the early diagnosis and treatment of pleural diseases [41]. Determining the most effective timing for the intervention can significantly impact patient outcomes. Research in this area can contribute to establishing evidence-based guidelines, optimizing the integration of medical thoracoscopy into clinical practice and enhancing its efficacy as a diagnostic and therapeutic tool.

Cost-effectiveness and comparative studies: Further research on the cost-effectiveness of different thoracoscopy approaches, including semi-rigid and rigid thoracoscopy, is crucial for refining medical thoracoscopy's diagnostic and therapeutic capabilities [10,42]. Comparative studies can provide valuable insights into techniques' relative advantages and disadvantages, informing clinicians and healthcare systems' decision-making. Understanding the economic implications of these approaches is essential for optimizing resource allocation and ensuring the sustainability of medical thoracoscopy in diverse healthcare settings.

Advances in pleural effusion diagnostics: Continuous investigation of the latest advances in the diagnosis of pleural effusion, based on the best available evidence, is vital for staying at the forefront of emerging technologies. Continuous research can provide valuable insights into novel diagnostic tools and approaches, their impact on diagnostic accuracy, and their potential to improve patient outcomes [43]. Keeping abreast of technological developments ensures that healthcare practitioners can leverage the most effective diagnostic modalities in the dynamic landscape of pleural effusion diagnostics.

Market growth and product trends: Continued research on the medical thoracoscopy market, including tracking product trends and new market developments, is essential for healthcare providers, researchers, and industry stakeholders [44]. Understanding market dynamics, including introducing new technologies and devices, can inform healthcare decisions, drive innovation, and ensure that the field evolves in response to the changing needs of patients and healthcare systems. Monitoring market trends is integral to fostering advancements in medical thoracoscopy and optimizing its accessibility and usability.

## Conclusions

In conclusion, examining medical thoracoscopy's role in pleural effusion assessment reveals several critical findings. Notably, its high diagnostic precision proves invaluable, especially when traditional modalities fail to provide conclusive results. Beyond its diagnostic capabilities, medical thoracoscopy introduces a therapeutic dimension, allowing for precise interventions like pleurodesis and targeted biopsies. However, the procedure has risks, underscoring the importance of careful patient selection and procedural expertise. Integrating medical thoracoscopy into clinical practice holds profound implications, promising enhanced diagnostic accuracy, tailored therapeutic approaches, and a heightened need for multidisciplinary collaboration. Its role emphasizes the importance of ongoing training and education to ensure proficiency among healthcare providers. As we navigate the dynamic landscape of respiratory medicine, the continued exploration of medical thoracoscopy is crucial, with the potential to refine protocols, improve safety profiles, and broaden its applicability in the management of pleural diseases. Thus, its place in routine clinical practice marks a promising stride toward more effective and personalized patient care in pleural effusion assessment.
